# Supplementary material for: Phage terminase recognition by the bacterial immune sensors Avs2 and Upx
Source: Nat Commun. 2026 Aug 3;17:10151. doi: 10.1038/s41467-026-76315-8 (PMC13601566; doi:10.1038/s41467-026-76315-8)
Supplement: Supplementary file 1 [file 41467_2026_76315_MOESM10_ESM.pdf]

nature portfolio

Peer Review File

Phage terminase recognition by the bacterial immune sensors Avs2 and Upx

Corresponding Author: Professor Alex Gao

This file contains all reviewer reports in order by version, followed by all author rebuttals in order by version.

Version 0:

Reviewer comments:

Reviewer #1

(Remarks to the Author)
This is a sound manuscript that reports a mechanism and structure of a novel prokaryotic Avs2 STAND NTPase (or the AVAST type 2 NTPase as reported in L. Gao et al 2020) in complex with a phage terminase it recognizes. The study begins with characterizing the activity of the AVAST defense systems against phage pathogen-associated molecular patterns (PAMPs) and their domains, particularly terminases, in a few iterations with genetic screens. In the assays, Avs2 showed high preference in activation to the C-terminal nuclease domain of terminases. Further, Avs2-PhiV-1 terminase complex was solved with cryoEM at a 2.2 A resolution. Interestingly, a new site with an ATP molecule was identified that stabilized the interface of EcAvs2 and terminase, which is distinct from the previously solved Avs3 structure. The protein defense activity was disrupted by mutating the residues binding this ATP molecule, indicating significance of this molecule for the complex function. Similarly, mutations disrupting electrostatic interactions in the Avs2-terminase binding interface revealed that multiple redundant interactions help recognizing the terminase fold.

In addition to Avs2, a novel Upx protein was shown to confer defense when co-expressed with a broad range of phage terminases. Interestingly, one bacterial clade of Upx was associated with terminase sensor Avs1, suggesting a functional relationship between the two systems.

In my opinion, this work will have a significance for the field of defense systems. It is following up on a new type of prokaryotic molecular pattern recognition mechanism characterized by A. Gao, F. Zhang and colleagues. The studied Avs2 protein is very distinct in sequence and structure identity to the previously characterized systems. Overall, the work has great quality structures, which are backed by mutational studies, and it supports the conclusions and claims it makes. There is nothing substantial I can request for the cryoEM part. Perhaps just a minor comment below could be addressed on the nuclease domain helix.

Line 128:
“To investigate the mechanism of terminase recognition by Avs2, we assembled the EcAvs2–PhiV-1 terminase complex in vitro in the presence of ATP and Mg2+ and found that it forms a stable complex with an approximately 1:1 stoichiometry following size-exclusion chromatography (Fig. 2a)”

It is not exactly clear how a 1:1 stoichiometry was derived, given that the intensity of the bands is different. This is not an issue for the manuscript story, however.

Line 156-159:
“However, we noticed cryo-EM density for a 10 amino-acid helix on the EcAvs2 TPR domain and could assign this density to a small fragment of the PhiV-1 terminase nuclease domain (Fig. 2g, magenta). Notably, these same amino acids are buried in the core of the nuclease domain in the SeAvs3-bound state of the PhiV-1 terminase. This observation suggests that EcAvs2 binding can partially unfold the terminase nuclease domain.”

That’s an interesting observation, but why is the conclusion about the terminase nuclease domain unfolding? Are ATPase and nuclease domains fixed rigidly in the terminase structure? Or does the distance not allow the helix to bind without unfolding? A clarification in the text would help the reader to understand the reason for this conclusion.

The conservation of the binding interface is believable ("the same amino acids buried in the core of the nuclease domain in the SeAvs3-bound state of the PhiV-1 terminase"). However, could you please show the fit of the model into your cryoEM maps for this region, possibly compared to Avs3, somewhere in the supplement?

Line 219-224:
"The structural basis by which terminase binding activates apo Avs2 remains to be elucidated. One possibility is that the multiple redundant interactions in the terminase binding interface contribute additively to a conformational rearrangement or rigidification of the TPR domain upon terminase binding, transmitting an allosteric signal to the ATPase that promotes oligomerization. Indeed, ligand binding-induced conformational activation of the NTPase domain is a common feature of STAND proteins, exemplified by the transcriptional regulator MalT and the plant resistosome protein ZAR1. "

I don't strictly request it, but I wondered if you would consider running a native gel/SEC to demonstrate the proposed oligomerization upon binding? I imagine it may be a part of future research, however.

Great job, and good luck with your research!

Reviewer #2

(Remarks to the Author)
Chiu et al. investigate the sensing mechanism of the antiviral STAND NTPase Avs2 and of Upx, an uncharacterized anti-phage defense system. Using an elegant co-expression toxicity assay, they show that diverse large terminase homologs are the principal triggers for E. coli and Klebsiella pneumoniae Avs2 (EcAvs2 and KpAvs2, respectively) as well as for Salmonella enterica Upx (SeUpx). Truncation analysis identifies the ATPase domain of the large terminase subunit as the key determinant of EcAvs2 and SeUpx activation—contrasting with SeAvs3, which can recognize either the ATPase or nuclease domain depending on the phage. The authors next solve a 2.2 Å cryo-EM structure of EcAvs2 bound to the PhiV-1 terminase, map the binding interface, and corroborate critical contacts by mutagenesis. Comparison with published structures of SeAvs3 and EcAvs4 highlights both shared and divergent recognition principles across the Avs family. Notably, the EcAvs2–PhiV-1 complex displays an extensive, partially redundant interface and a secondary "bridging ATP" molecule that may stabilize terminase engagement.

Overall, the study is systematic and technically rigorous. Nevertheless, because terminase was previously identified as the trigger for Avs proteins (Gao et al., Science, 2022), the conceptual advance of this study may be viewed as incremental. The following points should be addressed prior to publication:

Major issues:
1. The authors mutated a conserved phenylalanine in the EcAvs2 effector domain and found that the mutant no longer functions in anti-phage defense (Fig. S1). Do the authors believe this residue lies in a catalytic site? How can the authors be sure that the mutation does not affect folding or expression of the protein, both of which could account for the loss of protein function. Please provide evidence that the loss of activity is not due to mis-folding or poor expression (e.g., western blot).
2. Along the same lines as above, the authors mutate multiple residues that interact with the bridging ATP and found that a triple mutation (D981A/R1132A/E1364A) substantially disrupts anti-phage defense activity (Fig. 3C and S6A). Although these and other mutations in Fig. 3 disrupt defense, the possibility remains that some mutations simply destabilize the protein. Quantifying protein expression levels of these variants would strengthen the claim that the bridging ATP is mechanistically important.
3. The downstream effector activity of Avs2 remains unexplored. Any preliminary data—or even a concise discussion of feasible approaches—would substantially enhance the manuscript's impact.

Minor editorial issues:
1. Line 107: Define "TPR" on first use
2. Line 110: The figure citation should be to Fig. 1f.
3. Line 116: The figure citation should be to Fig. 1e.

Reviewer #3

(Remarks to the Author)
This manuscript investigates the specificity of Avs2-family abortive infection systems in bacteria, focusing on the molecular triggers that activate these defences during phage infection.
The barcode approach used by the authors to find other triggers other than terminases was very elegant. I really enjoyed reading it. Similarly, the structrure of the Avs-terminase complex that the authors solved nicely fits with their in vivo data and provides solid basis for the Avs activation mechanism. Thus, I have no further suggestion for the authors, as I feel their study is thorough and a great addition to the literature of the field.

Major comment:
1. Could the author expand a bit more on the rationale they used to choose specifically the Avs homologues shown in figure 1? Was it sequence diversity/niche? If the manuscript is focused on Avs2, why is Avs3 also used? Is the outlier control?
2. Can the authors explain better why, of the hundreds of defence systems that have been discovered, they decided to test

terminases vs upx? What was the rationale of the choice? That part of the paper is a bit disconnected from the rest and makes little sense in the way it's introduced.
3. Because Avs can recognise also Ksap proteins, could the authors perhaps model a complex between Ksaps and their new Avs structure to explore how these structural features change (or stay the same) when Avs is recognising Ksap.
4. The authors state that terminases are the main trigger, but I do not feel they tested enough Ksap1-2 homologues, compared to the terminases homologues to be able to confidently state that.
Minor comment:
1. Do the authors have a model for why Avs homologues from different recognise different pamps (KpAvs and EcAvs recognising Ksap proteins but not SeAvs)

Version 1:

Reviewer comments:

Reviewer #1

(Remarks to the Author)
The authors have done a great job on the manuscript and addressed all my comments. I recommend it for publication.

Reviewer #2

(Remarks to the Author)
The authors have sufficiently addressed my major concerns, and overall the revised manuscript has significantly improved since the previous version. I see no reason to delay publication any further.

Reviewer #3

(Remarks to the Author)
The authors have addressed all my comments in a satisfactory manner. No more comments from me.

Open Access This Peer Review File is licensed under a Creative Commons Attribution 4.0 International License, which permits use, sharing, adaptation, distribution and reproduction in any medium or format, as long as you give appropriate credit to the original author(s) and the source, provide a link to the Creative Commons license, and indicate if changes were made.
In cases where reviewers are anonymous, credit should be given to 'Anonymous Referee' and the source.
The images or other third party material in this Peer Review File are included in the article's Creative Commons license, unless indicated otherwise in a credit line to the material. If material is not included in the article's Creative Commons license and your intended use is not permitted by statutory regulation or exceeds the permitted use, you will need to obtain permission directly from the copyright holder.
To view a copy of this license, visit https://creativecommons.org/licenses/by/4.0/

Response to reviewers

We greatly appreciate the reviewers' thoughtful and constructive feedback, which has strengthened the study. We have incorporated the following major updates in the revised manuscript:

- Size exclusion chromatography analysis of EcAvs2 complex assembly, demonstrating that Avs2 oligomerization is induced by terminase binding (Fig. 1F)
- AlphaFold 3 models of diverse Upx–terminase complexes (ipTM ~0.8), which suggest an unexpected mode of target recognition where Upx binds to an unfolded state of the terminase ATPase domain
- Functional evaluation of two additional Upx homologs from *Hydrobacter penzbergensis* (HpUpx) and *Pyrinomonadaceae bacterium* (PbUpx), finding that they also detect terminases, which supports the conservation of this interaction across the Upx family
- Additional genetic screen results to ensure full coverage of ORFs from the *Dhillonvirus* Pal009, along with terminase truncations demonstrating that Upx senses the terminase ATPase domain
- A Western blot of EcAvs2 mutants to confirm stable protein expression

Reviewer #1

This is a sound manuscript that reports a mechanism and structure of a novel prokaryotic Avs2 STAND NTPase (or the AVAST type 2 NTPase as reported in L. Gao et al 2020) in complex with a phage terminase it recognizes. The study begins with characterizing the activity of the AVAST defense systems against phage pathogen-associated molecular patterns (PAMPs) and their domains, particularly terminases, in a few iterations with genetic screens. In the assays, Avs2 showed high preference in activation to the C-terminal nuclease domain of terminases. Further, Avs2-PhiV-1 terminase complex was solved with cryoEM at a 2.2 A resolution. Interestingly, a new site with an ATP molecule was identified that stabilized the interface of EcAvs2 and terminase, which is distinct from the previously solved Avs3 structure. The protein defense activity was disrupted by mutating the residues binding this ATP molecule, indicating significance of this molecule for the complex function. Similarly, mutations disrupting electrostatic interactions in the Avs2-terminase binding interface revealed that multiple redundant interactions help recognizing the terminase fold.

In addition to Avs2, a novel Upx protein was shown to confer defense when co-expressed with a broad range of phage terminases. Interestingly, one bacterial clade of Upx was associated with terminase sensor Avs1, suggesting a functional relationship between the two systems.

In my opinion, this work will have a significance for the field of defense systems. It is following up on a new type of prokaryotic molecular pattern recognition mechanism characterized by A. Gao, F. Zhang and colleagues. The studied Avs2 protein is very distinct in sequence and structure identity to the previously characterized systems. Overall, the work has great quality structures, which are backed by mutational studies, and it supports the conclusions and claims it makes. There is nothing substantial I can request for the cryoEM part. Perhaps just a minor comment below could be addressed on the nuclease domain helix.

We thank the reviewer for the positive and thoughtful feedback and have included point-by-point responses below.

Line 128:
"To investigate the mechanism of terminase recognition by Avs2, we assembled the EcAvs2–PhiV-1 terminase complex in vitro in the presence of ATP and Mg2+ and found that it forms a stable complex with an approximately 1:1 stoichiometry following size exclusion chromatography (Fig. 2a)"

It is not exactly clear how a 1:1 stoichiometry was derived, given that the intensity of the bands is different. This is not an issue for the manuscript story, however.

We thank the reviewer for raising this excellent point. We have revised the text to omit the mention of a 1:1 ratio in this section, as the stoichiometry of the complex is established by our subsequent cryo-EM structures:

(Lines 133-135): "To investigate the mechanism of terminase recognition by Avs2, we assembled an EcAvs2–PhiV-1 terminase complex *in vitro* in the presence of ATP and Mg^{2+} and purified a stable complex by SEC (Fig. 2a). We determined the structure of this complex using single-particle cryo-EM."

Line 156-159:
"However, we noticed cryo-EM density for a 10 amino-acid helix on the EcAvs2 TPR domain and could assign this density to a small fragment of the PhiV-1 terminase nuclease domain (Fig. 2g, magenta). Notably, these same amino acids are buried in the core of the nuclease domain in the SeAvs3-bound state of the PhiV-1 terminase. This observation suggests that EcAvs2 binding can partially unfold the terminase nuclease domain."

That's an interesting observation, but why is the conclusion about the terminase nuclease domain unfolding? Are ATPase and nuclease domains fixed rigidly in the terminase structure? Or does the distance not allow the helix to bind without unfolding? A clarification in the text would help the reader to understand the reason for this conclusion.

The conservation of the binding interface is believable ("the same amino acids buried in the core of the nuclease domain in the SeAvs3-bound state of the PhiV-1 terminase"). However, could you please show the fit of the model into your cryoEM maps for this region, possibly compared to Avs3, somewhere in the supplement?

We thank the reviewer for this insightful suggestion. The 11-amino-acid helix is normally buried within the hydrophobic core of the nuclease domain in its native, globular state, as observed in both the SeAvs3-bound structure and AlphaFold 3 predictions. However, in the EcAvs2–PhiV-1 terminase complex, the unfolded nuclease helix instead docks into a hydrophobic pocket on EcAvs2. If the helix remained part of the folded nuclease domain, structural superposition suggests that the rest of the nuclease would sterically clash with the EcAvs2 TPR sensor unless the nuclease domain were at least partially unfolded. To clarify our conclusion for nuclease domain unfolding, we have included additional views of the 11-amino-acid helix (residues 495–505) within our cryo-EM map (Fig. S5), and we have updated the text as follows:

(Lines 161–167): "However, we resolved a single 11-amino-acid helix from the nuclease domain lodged within a hydrophobic pocket on the EcAvs2 TPR domain (Fig. S5a,c–d and S8b). This interaction requires a large structural rearrangement of the natively folded terminase, as these residues are normally buried deep within the nuclease core (Fig. S5d), and superimposing the folded nuclease domain onto the EcAvs2-bound helix results in major steric clashes with the EcAvs2 TPR domain (Fig. S5e). These observations suggest that the terminase nuclease domain unfolds at least partially in order to bind to EcAvs2. This contrasts with SeAvs3, which extensively binds the terminase nuclease in its native conformation (Fig. 2d and Fig. S5b,d)."

Line 219-224:
"The structural basis by which terminase binding activates apo Avs2 remains to be elucidated. One possibility is that the multiple redundant interactions in the terminase binding interface contribute additively to a conformational rearrangement or rigidification of the TPR domain upon terminase binding, transmitting an allosteric signal to the ATPase that promotes oligomerization. Indeed, ligand binding-induced conformational activation of the NTPase domain is a common feature of STAND proteins, exemplified by the transcriptional regulator MalT and the plant resistosome protein ZAR1."

I don't strictly request it, but I wondered if you would consider running a native gel/SEC to demonstrate the proposed oligomerization upon binding? I imagine it may be a part of future research, however.

We thank the reviewer for the suggestion and have included a new SEC analysis of EcAvs2 complex assembly (Fig. 1f and S2), which demonstrated that EcAvs2 oligomerization is induced by terminase binding:

(Lines 128–131): "Finally, to investigate Avs2 activation biochemically, we analyzed recombinantly purified EcAvs2 and a representative terminase from phage T4 with size exclusion chromatography (SEC) in the presence of ATP and Mg^{2+}. A high molecular weight peak appeared only when both proteins were present, indicating that EcAvs2 oligomerization is induced by terminase binding (Fig. 1f and Fig. S2)."

Great job, and good luck with your research!

Reviewer #2

Chiu et al. investigate the sensing mechanism of the antiviral STAND NTPase Avs2 and of Upx, an uncharacterized anti-phage defense system. Using an elegant co-expression toxicity assay, they show that diverse large terminase homologs are the principal triggers for E. coli and Klebsiella pneumoniae Avs2 (EcAvs2 and KpAvs2, respectively) as well as for Salmonella enterica Upx (SeUpx). Truncation analysis identifies the ATPase domain of the large terminase subunit as the key determinant of EcAvs2 and SeUpx activation—contrasting with SeAvs3, which can recognize either the ATPase or nuclease domain depending on the phage. The authors next solve a 2.2 Å cryo-EM structure of EcAvs2 bound to the PhiV-1 terminase, map the binding interface, and corroborate critical contacts by mutagenesis. Comparison with published structures of SeAvs3 and EcAvs4 highlights both shared and divergent recognition principles across the Avs family. Notably, the EcAvs2–PhiV-1 complex displays an extensive, partially redundant interface and a secondary "bridging ATP" molecule that may stabilize terminase engagement.

Overall, the study is systematic and technically rigorous. Nevertheless, because terminase was previously identified as the trigger for Avs proteins (Gao et al., Science, 2022), the conceptual advance of this study may be viewed as incremental.

We greatly appreciate the reviewer's positive and thoughtful feedback. Beyond the structural characterization of the EcAvs2–terminase complex, our revised manuscript describes two novel mechanisms by which bacterial defense systems detect phage proteins: the utilization of a ligand to mediate an interaction interface, and the binding of partially unfolded target states. We have revised the discussion to better highlight these findings, which we believe represent a significant conceptual advance.

The following points should be addressed prior to publication:

Major issues:
1. The authors mutated a conserved phenylalanine in the EcAvs2 effector domain and found that the mutant no longer functions in anti-phage defense (Fig. S1). Do the authors believe this residue lies in a catalytic site? How can the authors be sure that the mutation does not affect folding or expression of the protein, both of which could account for the loss of protein function. Please provide evidence that the loss of activity is not due to mis-folding or poor expression (e.g., western blot).

We thank the reviewer for raising these important points and have performed Western blot analysis on the F67A mutant to assess its expression levels. Using standard Western Blotting ECL Substrate, no F67A band could be detected. A faint band was only observable upon utilizing SuperSignal West Femto Maximum Sensitivity ECL Substrate (see below), strongly suggesting that this mutation compromises protein expression or causes misfolding. As a result, we have removed the F67A results from Fig. S1 and the text for clarity.

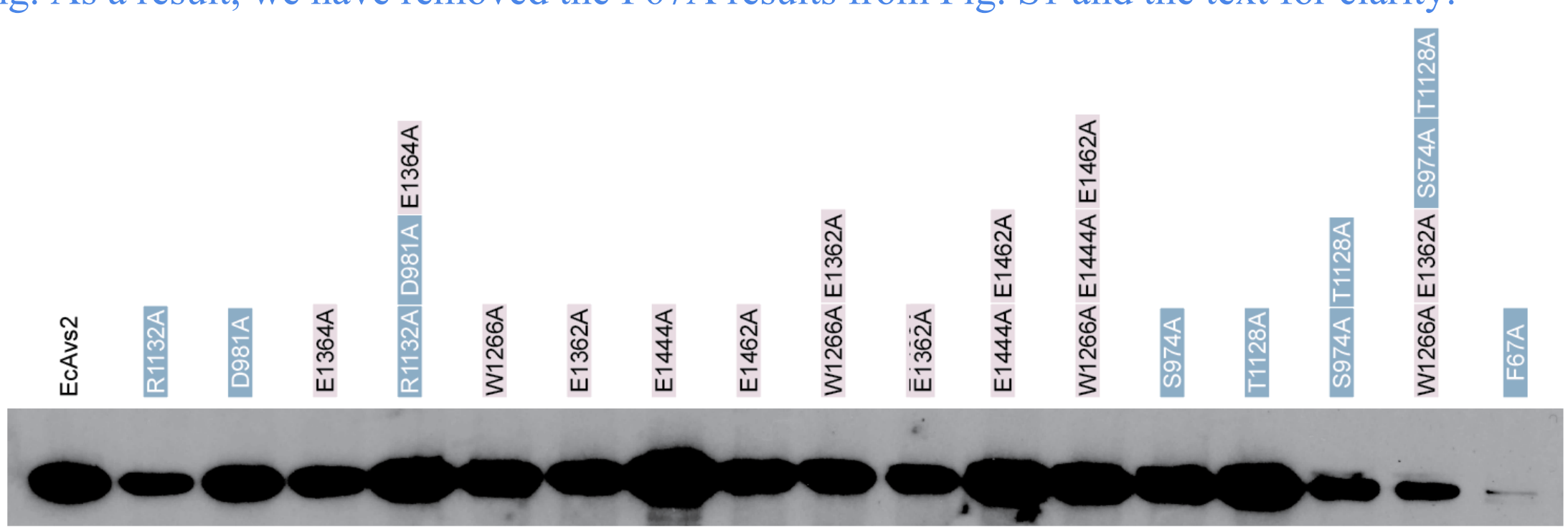

2. Along the same lines as above, the authors mutate multiple residues that interact with the bridging ATP and found that a triple mutation (D981A/R1132A/E1364A) substantially disrupts anti-phage defense activity (Fig. 3C and S6A). Although these and other mutations in Fig. 3 disrupt defense, the possibility remains that some mutations simply destabilize the protein. Quantifying protein expression levels of these variants would strengthen the claim that the bridging ATP is mechanistically important.

We thank the reviewer for raising this important point. We performed a Western blot on all mutants evaluated in Figure 3, including the bridging ATP triple mutant (D981A/R1132A/E1364A), demonstrating that they retain stable expression (Fig. S9).

3. The downstream effector activity of Avs2 remains unexplored. Any preliminary data—or even a concise discussion of feasible approaches—would substantially enhance the manuscript's impact.

We appreciate the helpful suggestion. As the N-terminal effector of EcAvs2 is uncommon within the Avs2 family, and our manuscript focuses on phage sensing, we believe a detailed investigation of the EcAvs2 effector is outside the scope of the current study, though it remains a compelling direction for future investigation. However, we note this effector domain is located at the N-terminus of at least eight additional defense systems (Fig. S6) and provide a multiple sequence alignment of the effector in the Supplementary Data. We have revised the text as suggested to discuss feasible approaches to investigate EcAvs2 effector function:

(Lines 243–251): "While most Avs2 homologs utilize Mrr-like dsDNA nucleases as effectors[3], the function of the less common EcAvs2 effector remains unknown. This domain appears to be highly modular, occurring at the N-terminus of at least eight other defense systems (Fig. S6a–b, Table S4). Its first 15 residues are homologous to amyloid signaling proteins involved in cell death[23], and its sequence contains conserved non-polar residues but lacks conserved charged or polar residues (see Supplementary Data), suggesting a non-enzymatic function. Consistent with this, we observed no cryo-EM density for the effector in the terminase-bound tetramer, indicating it is disordered or flexible in the absence of membranes or interacting partners. Further work such as affinity purification and mass spectrometry of EcAvs2 during phage infection, or the isolation of phage escape mutants and host mutants resistant to EcAvs2 toxicity, is required to determine its activity and the requirement for oligomerization."

Minor editorial issues:
1. Line 107: Define "TPR" on first use
2. Line 110: The figure citation should be to Fig. 1f.
3. Line 116: The figure citation should be to Fig. 1e.

We have updated the text and figures accordingly.

Reviewer #3

This manuscript investigates the specificity of Avs2-family abortive infection systems in bacteria, focusing on the molecular triggers that activate these defences during phage infection.
The barcode approach used by the authors to find other triggers other than terminases was very elegant. I really enjoyed reading it. Similarly, the structure of the Avs-terminase complex that the authors solved nicely fits with their in vivo data and provides solid basis for the Avs activation mechanism. Thus, I have no further suggestion for the authors, as I feel their study is thorough and a great addition to the literature of the field.

We greatly appreciate the reviewer's positive and thoughtful feedback and have included point-by-point responses below.

Major comment:
1. Could the author expand a bit more on the rationale they used to choose specifically the Avs homologues shown in figure 1? Was it sequence diversity/niche? If the manuscript is focused on Avs2, why is Avs3 also used? Is the outlier control?

We thank the reviewer for raising this important point. We focused on the structural characterization of Avs2 because AlphaFold modeling indicated it is structurally distinct from the other known terminase sensors, Avs1 and Avs3. Among Avs2 homologs, we selected EcAvs2 as a model because of its established activity in plaque assays against diverse phages (Gao et al. 2020). SeAvs3 served as a control in our coexpression assays, as both EcAvs2 and SeAvs3 defend against phages PhiV-1 and ZL19 and are toxic when co-expressed with their terminases (Gao et al. 2022), facilitating comparisons between these two systems. We have revised the text as follows:

(Lines 70–73): "While Avs1 shares strong predicted structural similarity with Avs3, Avs2 is predicted to be structurally distinct, and how it recognizes terminases remains unknown. Avs2 is also the most widespread and abundant of these systems, with extensive horizontal gene transfer and effector domain diversity[3]."

(Lines 87–92): "We previously showed that EcAvs2 confers defense against diverse types of tailed phages, including the T7-like phage PhiV-1 (ref.[17]) and the T1-like *Drexlerviridae* phage ZL19 (ref.[3]). We screened for EcAvs2 activators across the entire proteomes of these two phages using a co-expression toxicity assay, in which the expression of EcAvs2 with its cognate trigger in the same bacterial cell leads to EcAvs2-mediated cell death[8,17] (Fig. 1a and Table S1). SeAvs3 was included in parallel as a control, as it confers defense against both PhiV-1 and ZL19 and is activated only by the terminase in each genome[3]."

2. Can the authors explain better why, of the hundreds of defence systems that have been discovered, they decided to test terminases vs Upx? What was the rationale of the choice? That part of the paper is a bit disconnected from the rest and makes little sense in the way it's introduced.

We thank the reviewer for the excellent suggestion. The large sensor domain of Upx led us to hypothesize that it might recognize a phage protein. To test this hypothesis, we co-expressed Upx with each gene in the PhiV-1 genome and identified gp19 terminase as the sole trigger of SeUpx (Fig. 4 and Fig. S12–14). To assess the generalizability of these findings, we cloned divergent Upx homologs from *Hydrobacter penzbergensis* (HpUpx) and *Pyrinomonadaceae bacterium* (PbUpx) (Fig. 4a), finding that both induce cellular toxicity when co-expressed with diverse terminases (Fig. 4d).

We re-organized the Upx findings into separate figures (Fig. 4 and S12–14) and dedicated a separate results

section for Upx.

(Lines 74–78): "In addition, it is unclear whether broad-specificity terminase recognition is unique to STAND-based sensors or shared by other defense systems. One enigmatic candidate is the defense protein Upx[17], which contains a large unannotated sensor domain and a PD-(D/E)xK nuclease domain but lacks a STAND NTPase. Although evolutionarily distinct from Avs proteins, the size of the Upx sensor suggests it may similarly recognize phage proteins. However, the specific triggers and the mechanism of Upx activation during infection remain unknown."

(Line 203 section header): "The defense protein Upx is a terminase sensor distinct from Avs1–3"

3. Because Avs can recognise also Ksap proteins, could the authors perhaps model a complex between Ksaps and their new Avs structure to explore how these structural features change (or stay the same) when Avs is recognising Ksap.

We appreciate this excellent suggestion and have included new structural comparisons of the Avs2–terminase and Avs2–Ksap1/2 complexes (Fig. S10–11). Consistent with previous observations (Béchon et al., 2024), Ksap1 and Ksap2 are predicted to bind the same pocket on Avs2 as the terminase, but the Avs2–terminase interface is more extensive, with larger buried surface area (e.g., 2833.5 Å^2 for EcAvs2–terminase vs. 1573.6 Å^2 for EcAvs2–Ksap1). We also detected structural similarity between the terminase ATPase domain and both Ksap1 and Ksap2. We have revised the text as follows:

(Lines 189–194): "Although Ksap1 and Ksap2 are predicted to bind the same pocket of Avs2 as the PhiV-1 terminase, they interact primarily with the CTD sensor rather than the more extensive binding interface utilized by the terminase (Fig. 2c and Fig. S10). Consistent with this observation, the predicted buried surface area is substantially larger for Avs2–terminase than for Avs2–Ksap1/2 (e.g., 2833.5 Å^2 for EcAvs2–terminase vs. 1573.6 Å^2 for EcAvs2–Ksap1). Nevertheless, Ksap1 and Ksap2 adopt secondary structures similar to the terminase, featuring a central 4–6-stranded β-sheet and flanking helices that face the CTD sensor (Fig. S11)."

4. The authors state that terminases are the main trigger, but I do not feel they tested enough Ksap1-2 homologues, compared to the terminases homologues to be able to confidently state that.

We thank the reviewer for raising this important point. While we tested 37 different terminases compared to two homologs each of Ksap1 and Ksap2, this difference in sampling was not due to experimental bias. Rather, Ksap1 and Ksap2 homologs exhibit substantially less sequence diversity because they are restricted to specific phage lineages. In contrast, terminases are universal across tailed phages, with homologs from different phage families exhibiting extreme sequence divergence. More importantly, our hypothesis that the terminase is the canonical trigger of Avs2, with non-terminase activators representing intriguing lineage-specific exceptions, is based primarily on structural and evolutionary considerations. We have clarified the discussion as follows:

(Lines 252–262): "Although non-terminase activators such as Ksap1, Ksap2, and possibly vs.8 are intriguing, several lines of evidence suggest that the terminase is the primary evolutionary target of Avs2. The Avs2–terminase complex is defined by strong shape and charge complementarity between the Avs2 sensor and the terminase. While Ksap1, Ksap2, and vs.8 share structural similarity with the terminase ATPase and are predicted to bind the Avs2 pocket in the same orientation, they do not engage the full binding interface, suggesting that Avs2 is adapted for the terminase. Consistent with this hypothesis, Avs2 appears to have a secondary binding interface that can in some cases interact with the terminase nuclease domain, an interaction that is absent with the three non-terminase activators. Moreover, Ksap1, Ksap2, and vs.8 are restricted to

specific phage taxa, whereas the terminase is universal across tailed phages, consistent with the broad anti-phage specificity of Avs2 and its presence across more than 20 prokaryotic phyla[3]. Together, these observations support a model in which the terminase is the canonical trigger of Avs2, with non-terminase activators representing lineage-specific exceptions."

Minor comment:
1. Do the authors have a model for why Avs homologues from different recognise different PAMPs (KpAvs and EcAvs recognising Ksap proteins but not SeAvs)

We appreciate this interesting point. We believe these differences in PAMP recognition arise from the extensive sequence and structural variation present in both the Avs proteins (e.g., within the TPR repeats) and their diverse phage-derived triggers. For instance, homologs of Avs2, as well as those of terminases, frequently share <10% sequence identity. Consequently, these systems rely on the recognition of conserved structural features, a hypothesis consistent with the structural similarity between Ksap1, Ksap2, vs.8, and the terminase ATPase domains. Because of this variation, the activation of oligomerization for specific Avs–trigger pairs remains difficult to predict with current methods. Just as members of a given Avs family do not all recognize the same homologs of its phage protein target, it is expected that they also do not universally recognize the same Ksap proteins. This effect is perhaps even more pronounced across different Avs families. We agree that the existence of non-canonical triggers for other Avs families is an intriguing question for further investigation.
