# Supplementary material for: Phage terminase recognition by the bacterial immune sensors Avs2 and Upx
Source: Nat Commun. 2026 Aug 3;17:10151. doi: 10.1038/s41467-026-76315-8 (PMC13601566; doi:10.1038/s41467-026-76315-8)
Supplement: Supplementary file 3 [file 41467_2026_76315_MOESM2_ESM.pdf]

Description of Additional Supplementary Files

File name: Supplementary Data 1
Description: Defense systems in this study.

File name: Supplementary Data 2
Description: AlphaFold 3 statistics for toxic proteins from PhiV-1 and ZL19 co-folded with defense proteins.

File name: Supplementary Data 3
Description: Terminase, Ksap1/2, and vs.8 homologs tested in toxicity screens.

File name: Supplementary Data 4
Description: List of proteins in GenBank containing the EcAvs2 effector domain.

File name: Supplementary Data 5
Description: List of Upx homologs in GenBank.

File name: Supplementary Data 6
Description: AlphaFold 3 models in this study.
