# Supplementary material for: Phage terminase recognition by the bacterial immune sensors Avs2 and Upx
Source: Nat Commun. 2026 Aug 3;17:10151. doi: 10.1038/s41467-026-76315-8 (PMC13601566; doi:10.1038/s41467-026-76315-8)
Supplement: Supplementary file 4 [file 41467_2026_76315_MOESM9_ESM.pdf]

nature portfolio

Corresponding author(s): Alex Gao

Last updated by author(s): 6/30/2026

Reporting Summary

Nature Portfolio wishes to improve the reproducibility of the work that we publish. This form provides structure for consistency and transparency in reporting. For further information on Nature Portfolio policies, see our Editorial Policies and the Editorial Policy Checklist.

Statistics

For all statistical analyses, confirm that the following items are present in the figure legend, table legend, main text, or Methods section.

n/a	Confirmed	
☐	☒	The exact sample size (n) for each experimental group/condition, given as a discrete number and unit of measurement
☒	☐	A statement on whether measurements were taken from distinct samples or whether the same sample was measured repeatedly
☒	☐	The statistical test(s) used AND whether they are one- or two-sided<br>*Only common tests should be described solely by name; describe more complex techniques in the Methods section.*
☒	☐	A description of all covariates tested
☒	☐	A description of any assumptions or corrections, such as tests of normality and adjustment for multiple comparisons
☐	☒	A full description of the statistical parameters including central tendency (e.g. means) or other basic estimates (e.g. regression coefficient) AND variation (e.g. standard deviation) or associated estimates of uncertainty (e.g. confidence intervals)
☒	☐	For null hypothesis testing, the test statistic (e.g. F, t, r) with confidence intervals, effect sizes, degrees of freedom and P value noted<br>*Give P values as exact values whenever suitable.*
☒	☐	For Bayesian analysis, information on the choice of priors and Markov chain Monte Carlo settings
☒	☐	For hierarchical and complex designs, identification of the appropriate level for tests and full reporting of outcomes
☒	☐	Estimates of effect sizes (e.g. Cohen's d, Pearson's r), indicating how they were calculated

Our web collection on statistics for biologists contains articles on many of the points above.

Software and code

Policy information about availability of computer code

Data collection	Cryo-EM: EPU (Thermo Fisher Scientific; AFIS mode) was used to acquire cryo-EM movies on a Titan Krios G3i with a Gatan K3 detector.
Data analysis	Prokka (v1.14.6), ChimeraX (v1.8), PyMOL (v2.5.7 and v3.0.3), PSI-BLAST, AlphaFold3, AlphaFold2, ImageJ, PROMALS3D, MATLAB 2024a, Adobe Illustrator (2024, 2025, and 2026), iTOL (v7), MAFFT (v7.520), MMSeqs2, trimAl v1.2, IQ-TREE (v1.6.2), RPSBLAST, RELION, ISOLDE, PHENIX

For manuscripts utilizing custom algorithms or software that are central to the research but not yet described in published literature, software must be made available to editors and reviewers. We strongly encourage code deposition in a community repository (e.g. GitHub). See the Nature Portfolio guidelines for submitting code & software for further information.

Data

Policy information about availability of data

All manuscripts must include a data availability statement. This statement should provide the following information, where applicable:

- Accession codes, unique identifiers, or web links for publicly available datasets
- A description of any restrictions on data availability
- For clinical datasets or third party data, please ensure that the statement adheres to our policy

Cryo-EM maps have been deposited in the Electron Microscopy Data Bank with accession codes EMD-70096 and EMD-70097. The corresponding atomic model has been deposited in the Protein Data Bank under the accession code 9O4E.

Research involving human participants, their data, or biological material

Policy information about studies with human participants or human data. See also policy information about sex, gender (identity/presentation), and sexual orientation and race, ethnicity and racism.

Reporting on sex and gender	NA
Reporting on race, ethnicity, or other socially relevant groupings	NA
Population characteristics	NA
Recruitment	NA
Ethics oversight	NA

Note that full information on the approval of the study protocol must also be provided in the manuscript.

Field-specific reporting

Please select the one below that is the best fit for your research. If you are not sure, read the appropriate sections before making your selection.

☒ Life sciences ☐ Behavioural & social sciences ☐ Ecological, evolutionary & environmental sciences

For a reference copy of the document with all sections, see nature.com/documents/nr-reporting-summary-flat.pdf

Life sciences study design

All studies must disclose on these points even when the disclosure is negative.

Sample size	No statistical methods were used to predetermine sample size. For cryo-EM studies, data size was determined by availability of instrument time and the relative particle density on cryo-EM grids.
Data exclusions	We omitted the EcAvs2 F67A mutant as the band was not visible in Western blots, suggesting the mutant compromises protein expression or causes misfolding.
Replication	Co-expression toxicity assays and plaque assays were repeated at least three times in independent experiments, with reliable results.
Randomization	Randomization was not applicable. For each experiment, all conditions/mutants were treated the same.
Blinding	Blinding was not relevant to the experiments in our study since no subjective allocation was involved.

Reporting for specific materials, systems and methods

We require information from authors about some types of materials, experimental systems and methods used in many studies. Here, indicate whether each material, system or method listed is relevant to your study. If you are not sure if a list item applies to your research, read the appropriate section before selecting a response.

Materials & experimental systems

n/a	Involved in the study	
☐	☒	Antibodies
☒	☐	Eukaryotic cell lines
☒	☐	Palaeontology and archaeology
☒	☐	Animals and other organisms
☒	☐	Clinical data
☒	☐	Dual use research of concern
☒	☐	Plants

Methods

n/a	Involved in the study	
☒	☐	ChIP-seq
☒	☐	Flow cytometry
☒	☐	MRI-based neuroimaging

Antibodies

Antibodies used	Strep-Tag II monoclonal antibody (1810CT579.47.56.10) (Invitrogen; Ref: MA5-37747; Lot: 79994304)<br>Peroxidase-conjugated AffiniPure Donkey Anti-Mouse IgG (Jackson ImmunoResearch; Code 715-035-150; Lot: 177916)

Validation	Strep-Tag II monoclonal antibody validation: https://www.thermofisher.com/antibody/product/Strep-Tag-II-Antibody-clone-1810CT579-47-56-10-Monoclonal/MA5-37747

Plants

Seed stocks	NA
Novel plant genotypes	NA
Authentication	NA
